# Vulvovaginal Trichosporonosis

**DOI:** 10.1080/10647440300025510

**Published:** 2003

**Authors:** Paul Makela, Debbie Leaman, Jack D. Sobel

**Affiliations:** 1 Department of Obstetrics and Gynecology Wayne State University School of Medicine 4707 St Antoine Detroit MI 48201 USA; 2 Division of Infectious Disease Wayne State University School of Medicine Detroit MI USA

## Abstract

*Objective:* Isolation of *Trichosporon* species from vaginal secretions is a rare event, and no data are available on its pathogenic role. A case series is presented to determine the pathogenic role of *Trichosporon* species in vulvovaginal infections.

*Methods:* We performed a retrospective chart review of patients seen in the W.S.U. Vaginitis Clinic in order to identify patients from whom *Trichosporon*  species were isolated.

*Results:* Between 1986 and 2001, a total of 13 patients had a total of 18 positive vaginal cultures for *Trichosporon* species. All 18 vaginal isolates were *T. inkin*. In general, positive vaginal cultures were accompanied by low yeast colony counts. Four out of 18 positive *T. inkin* cultures were obtained from visits by asymptomatic patients. Of the remaining 14 positive *T. inkin* cultures from patients with symptoms, nine out of 14 cultures had other diagnoses (*Candida albicans*, six cases; bacterial vaginosis, two cases; *Trichomonas*, one case). Five positive *T. inkin* cultures were obtained from visits at which patients had symptoms and no associated diagnosis. In only one of the five episodes could we establish a clear pathogenic role for *Trichosporon*. In this case the patient was treated with boric acid and had resolution of symptoms and a negative culture at follow-up. *In-vitro* susceptibility tests revealed that *T. inkin* was resistant to flucytosine and susceptible to all topical and oral azoles.

*Conclusions:* *T. inkin* is occasionally found in vulvovaginal cultures and is usually a non-pathogen. Transient colonization tended to occur in women, usually of African—American origin, with major perturbations in vaginal flora (bacterial vaginosis and trichomoniasis) and increased pH. Pathogenic consequences of *Trichosporon* colonization appear to be rare.


*Infect Dis Obstet Gynecol 2003;11:131–133*

### Vulvovaginal trichosporonosis

**Paul Makela[1], Debbie Leaman[2] and Jack D. Sobel[2]**

[1]*Department of Obstetrics and Gynecology and*
[2]*Division of Infectious Disease, Wayne State University School of Medicine, Detroit, MI*

***Objective:*** Isolation of *Trichosporon* species from vaginal secretions is a rare event, and no data are available on its pathogenic role. A case series is presented to determine the pathogenic role of *Trichosporon* species in vulvovaginal infections.
***Methods:*** We performed a retrospective chart review of patients seen in the W.S.U. Vaginitis Clinic in order to identify patients from whom *Trichosporon* species were isolated.
***Results:*** Between 1986 and 2001, a total of 13 patients had a total of 18 positive vaginal cultures for *Trichosporon* species. All 18 vaginal isolates were *T. inkin*. In general, positive vaginal cultures were accompanied by low yeast colony counts. Four out of 18 positive *T. inkin* cultures were obtained from visits by asymptomatic patients. Of the remaining 14 positive *T. inkin* cultures from patients with symptoms, nine out of 14 cultures had other diagnoses (*Candida albicans*, six cases; bacterial vaginosis, two cases; *Trichomonas*, one case). Five positive *T. inkin* cultures were obtained from visits at which patients had symptoms and no associated diagnosis. In only one of the five episodes could we establish a clear pathogenic role for *Trichosporon*. In this case the patient was treated with boric acid and had resolution of symptoms and a negative culture at follow-up. *In-vitro* susceptibility tests revealed that *T. inkin* was resistant to flucytosine and susceptible to all topical and oral azoles.
***Conclusions:*** *T. inkin* is occasionally found in vulvovaginal cultures and is usually a non-pathogen. Transient colonization tended to occur in women, usually of African–American origin, with major perturbations in vaginal flora (bacterial vaginosis and trichomoniasis) and increased pH. Pathogenic consequences of *Trichosporon* colonization appear to be rare.

Key words: *TRICHOSPORON INKIN*; VAGINITIS; BACTERIAL VAGINOSIS; VULVOVAGINAL CANDIDIASIS

---

*Trichosporon* is a genus of basidiomycetous yeasts. *Trichosporon* species are widely distributed, being found in soil, stagnant and fresh water and animal excrement[1]. They may also be part of the normal human skin and respiratory tract flora[1]. In 1992, the genus *Trichosporon* was revised by Gueho *et al*.[2]. Six human pathogenic species of *Trichosporon* have been described, namely *T. asahii*, *T. asteroids*, *T. cutaneum*, *T. inkin*, *T. mucoides* and *T. ovoids*[3]. A well-known manifestation of *Trichosporon* infection is white piedra, with the formation of hard nodules on hair shafts[4]. *Trichosporon* species also commonly cause cutaneous infections[5]. Increasingly, *Trichosporon* species may be involved in invasive local or disseminated infections in immunocompromised patients, often with a fulminant course and fatal outcome[1].

Specifically, *T. inkin* is known to be involved in the occurrence of white piedra in the genital area[4]. Other manifestations of *T. inkin* infection that have been reported include prosthetic mitral valve endocarditis[6], peritonitis in a peritoneal dialysis patient[7], and a pulmonary abscess in a child with chronic granulomatous disease[8]. Isolation of

Correspondence to: Paul Makela, MD, 4707 St Antoine, Detroit, MI 48201, USA. Email: pmakela@med.wayne.edu

© 2003 The Parthenon Publishing Group 

*Trichosporon* species from the vaginal canal is a rare event, and no data are available on its pathogenic role.

### SUBJECTS AND METHODS

### Study population

The Vaginitis Clinic laboratory records were reviewed for all cultures growing *Trichosporon* species from 1986 to 2002. A total of 13 patients had positive vaginal cultures for *Trichosporon* species.

### Laboratory methods

All vaginal specimens of patients who are seen in the clinic, whether those individuals are symptomatic or not, are cultured for fungi. Specimens were placed on plates with media selective for *Candida* growth (Sabouraud's dextrose agar). Suspected mixed cultures, as determined by colony type and macroscopic characteristics, were placed on plates with media showing species-specific colony color change (Chromagar, CHROMagar, Paris, France). *Trichosporon* species were identified by carbohydrate assimilation tests (API-20C Aux). *In-vitro* susceptibility tests were performed using the National Committee for Clinical Laboratory Standards M27-A method[9].

### RESULTS

A total of 13 patients who were seen at the Vaginitis Clinic between 1986 and 2002 had a total of 18 positive cultures for *Trichosporon* species. Notably, 11 out of 13 patients (85%) were of African–American origin. The 13 patients had a mean age of 32 years. One patient had a history of diabetes mellitus. All 18 vaginal isolates were identified as *T. inkin*. In general, positive *Trichosporon* vaginal cultures were accompanied by low yeast colony counts. In addition to two women with bacterial vaginosis and one with trichomoniasis, six positive *T. inkin* cultures were obtained from women with an elevated pH (pH > 4.5). Routine saline and 10% potassium hydroxide microscopy were rarely positive for yeast and could not determine a pathogenic role for *T. inkin*. Four out of 18 positive *T. inkin* cultures were obtained from visits by three asymptomatic patients who were seen on routine follow-up examinations. Of the remaining 14 positive *T. inkin* cultures obtained from patients with symptoms, nine out of 14 cultures had other diagnostic entities or pathogens, namely *Candida albicans* ($n = 6$), bacterial vaginosis ($n = 2$) and trichomoniasis ($n = 1$). Five positive *T. inkin* cultures were obtained from visits at which patients had symptoms and no associated diagnosis. Two out of five positive *T. inkin* cultures were obtained from patients who had no follow-up. One out of five positive *T. inkin* cultures was obtained from a patient who received no treatment and had a negative culture at the next visit, and one out of five positive *T. inkin* cultures was obtained from a patient who was not treated until a follow-up visit, which was culture positive for *T. inkin* and *C. albicans*. The patient showed resolution of symptoms and had a negative culture after treatment with fluconazole. Only one out of five positive *T. inkin* cultures was obtained from a patient who had vulvovaginal symptoms and no other diagnostic etiology apart from *T. inkin*. This patient showed resolution of symptoms and negative cultures with topical boric acid therapy, 600 mg daily for 14 days. This was the only patient in whom we could establish a probable pathogenic role for *Trichosporon*. *In-vitro* susceptibility tests revealed that *T. inkin* was resistant to flucytosine and susceptible to all topical and oral azoles. Testing of the susceptibility of *Trichosporon* species to azoles revealed the following minimal inhibitory concentration (MIC) ranges: fluconazole, 1–4 mg/ml; intraconazole, 0.125–0.5 mg/ml; voriconazole, 0.3–0.125 mg/ml; miconazole, 0.03–0.5 mg/ml; clotrimazole, 0.3–0.125 mg/ml; ketoconazole, 0.06–0.25 mg/ml.

### DISCUSSION

Vulvovaginal candidiasis (VVC) is the second most common cause of vaginal infections after bacterial vaginosis in the USA. If only US studies are considered, 80–95% of VVC is caused by *Candida albicans* and the rest is caused by a variety of non-*albicans Candida* species[10]. Less than 1% of vaginal fungal infections are caused by non-*Candida* species[11]. Unusual fungi that have been reported include *Saccharomyces cervisiae*[12] and *Zygomycetes*[13], which were reported in healthy non-immunocompromised patients. Although it is known that *Trichosporon* species infrequently cause invasive infections in humans, there is no report in the literature of vaginitis caused by any species of *Trichosporon*. Based on our analysis, *T. inkin* is occasionally found in vulvovaginal cultures and is usually a non-pathogen. *Trichosporon* species (e.g. *T. asahii*) commonly cause cutaneous and invasive systemic disease, whereas *T. inkin* appears to be selectively and uniquely isolated from genital specimens[5,14]. In only one out of 13 patients could a likely pathogenic role be established for *T. inkin*. In this patient, elimination of vulvovaginal symptoms corresponded to previously positive vaginal cultures for *T. inkin* becoming negative following antimycotic therapy. Transient colonization tended to occur predominantly in African–American women with major perturbations of the vaginal flora (bacterial vaginosis and trichomoniasis) with increased vaginal pH. Pathogenic consequences of *Trichosporon* colonization appear to be extremely rare. Treatment of *Trichosporon* should be withheld until a second culture reveals persistence of *T. inkin* in a symptomatic patient in whom no other cause of symptoms is identified. Based on *in-vitro* susceptibility, clinical eradication of *T. inkin* should follow treatment with topical or oral azoles.

### REFERENCES

1. Itoh T, Hosokawa H, Kohdera U, *et al.* Disseminated infection with *Trichosporon asahii*. *Mycoses* 1996;39:195–9
2. Gueho E, Smith MTH, deHoog GS, *et al.* Contributions to a revision of the genus *Trichosporon*. *Antonie Van Leeuwenhoek* 1992;61:289–316
3. Gueho E, Improvisi L, deHoog GS, *et al. Trichosporon* on humans: a practical account. *Mycoses* 1994;37:3–10
4. Therizol-Ferly M, Kombila M, Gomez de Diaz M, *et al. Piedra* and *Trichosporon* species in equatorial Africa. *Mycoses* 1994;37:249–53
5. Mayser P, Huppertz M, Papavassilis CH, *et al.* Yeasts of the genus *Trichosporon*. Identification, epidemiology and significance in dermatological disease. *Hautarzt* 1996;47:913–20
6. Chaumentin G, Boibeux A, Piens MA, *et al. Trichosporon inkin* endocarditis; short-term evolution and clinical report. *Clin Infect Dis* 1996; 23:396–7
7. Lopes JO, Alves SH, Klock C, *et al. Trichosporon inkin* peritonitis during continuous ambulatory peritoneal dialysis with bibliography review. *Mycopathologia* 1997;139:15–18
8. Piwoz JA, Stadtmauer GJ, Bottone EJ, *et al. Trichosporon inkin* lung abscesses presenting as a penetrating chest mass. *Pediatr Infect Dis J* 2000;19:1025–7
9. National Committee for Clinical Laboratory Standards. *Development of* In Vitro *Susceptibility Testing Criteria and Quality Control Parameters. Approved Guidelines for M27-A*. Villanova, PA; National Committee for Laboratory Standards, 1994
10. Odds FC. Candidosis of the genitalia. In *Candida* and *Candidiasis. A Review and Bibliography*, 2nd edn. Philadelphia, PA: Bailliere Tindall 1998:124–36
11. Sobel JD. Epidemiology and pathogenesis of recurrent vulvovaginal *candidiasis*. *Am J Obstet Gynecol* 1985;152:924–35
12. Sobel J, Vazquez J, Lynch M, *et al.* Vaginitis due to *Saccharomyces cervisiae*. Epidemiology, clinical aspects and therapy. *Clin Infect Dis* 1993;16:93–9
13. Sobel JD. Vaginal mucomycosis: a case report. *Infect Dis Obstet Gynecol* 2001;9:117–18
14. Ebright JR, Fairfax MR, Vazquez JA. *Trichosporon asahii*, a non-*Candida* yeast that caused fatal septic shock in a patient without cancer or neutropenia. *Clin Infect Dis* 2001;33:28–30

Received 09/16/02; Accepted 03/17/03
